# Results of a Multidisciplinary Stepwise Protocol to Treat Chronic Refractory Kidney-Related Pain

**DOI:** 10.3390/jcm14165623

**Published:** 2025-08-08

**Authors:** Paul Geertsema, Ron T. Gansevoort, Benjamin H. J. Doornweerd, Robbert J. de Haas, Joke M. Perdok, Stijn Roemeling, Ruud Stellema, André P. Wolff, Niek F. Casteleijn

**Affiliations:** 1 Department of Nephrology, Expertise Center for Polycystic Diseases, University Medical Center Groningen, University of Groningen, P.O. Box 30.001, 9700 RB Groningen, The Netherlands; r.t.gansevoort@umcg.nl; 2 Department of Urology, University Medical Center Groningen, University of Groningen, P.O. Box 30.001, 9700 RB Groningen, The Netherlands; b.h.j.doornweerd@umcg.nl (B.H.J.D.); s.roemeling@umcg.nl (S.R.); n.f.casteleijn@umcg.nl (N.F.C.); 3 Department of Radiology, University Medical Center Groningen, University of Groningen, P.O. Box 30.001, 9700 RB Groningen, The Netherlands; r.j.de.haas@umcg.nl; 4 Department of Anesthesiology, University Medical Center Groningen, University of Groningen, P.O. Box 30.001, 9700 RB Groningen, The Netherlands; j.m.perdok@umcg.nl (J.M.P.); r.stellema@umcg.nl (R.S.); a.p.wolff@umcg.nl (A.P.W.); 5 Department of Urology, Ommelander Ziekenhuis, 9679 BJ Scheemda, The Netherlands

**Keywords:** pain, treatment, nerve blocks, kidney disease, kidney-related pain

## Abstract

**Background**: Kidney-related pain can be chronic, disabling and negatively impact quality of life. In this prospective case series, we assessed whether a stepwise multidisciplinary treatment protocol, originally developed to treat ADPKD-related pain, can provide significant pain relief in non-ADPKD patients with kidney-related pain. **Methods:** Patients were eligible if they had incapacitating kidney-related pain with a visual analogue scale (VAS) score ≥50 out of 100, lasting ≥3 months and with insufficient response to previous treatments. The main exclusion criterion was ADPKD. Treatment options were, in order when indicated, nonpharmacological treatments, analgesics, cyst aspiration and fenestration, nerve blocks and nephrectomy. The effect of treatment on pain was investigated by means of VAS scores, defined daily dose of pain medication and quality-of-life scores. **Results:** Twelve patients (67% female, median age 50 [IQR: 36–59] years), with a median duration of pain of 1.9 [1.0–4.7] years, were included. In 50% of cases, chronic pain remained after an acute episode of kidney stones. Median follow-up after treatment was 3.8 [IQR: 2.5–4.4] years. The VAS before treatment (70 (48–90)) was reduced at short-term (35 [28–53], *p* = 0.01) and long-term follow-up (40 [38–53], *p* = 0.01). In addition, the defined daily dose of both opioids and non-opioids was reduced at short-term follow-up (*p* = 0.04 and 0.04, respectively) as well as at long-term follow-up (*p* = 0.03 and *p* = 0.02, respectively). **Conclusions:** We found that our multidisciplinary treatment protocol is effective in achieving sustained pain relief as well as a reduction in the use of pain medication in non-ADPKD patients with chronic, refractory kidney-related pain.

## 1. Introduction

Loin pain is a common symptom in the general population and is kidney-related in a substantial number of patients. The prevalence of loin pain is not exactly known but is estimated to be around 10% in the general population [1]. It can be arbitrarily categorized as acute vs. chronic loin pain when it exists for >3 months. Acute kidney-related loin pain can have a lot of different possible causes, such as kidney stones, upper urinary tract infection or trauma, while chronic kidney-related loin pain, among other things, can be caused by anatomic abnormalities such as ureteropelvic junction (UPJ) stenosis, horseshoe kidney, parapelvic kidney cysts or retroperitoneal fibrosis but can also develop after an acute pain-causing event due to sensitization [2,3,4,5,6,7]. While some patients are reported to have spontaneous remission after a couple of years, a significant number of patients have chronic debilitating pain [8]. It is known that chronic kidney-related pain negatively influences quality of life and is often difficult to manage [9].

In genetic disorders such as autosomal dominant polycystic kidney disease (ADPKD), chronic pain is a common symptom due to the enlargement of the kidneys [10]. In the last decade, our expertise center for polycystic kidney disease developed and evaluated a multidisciplinary stepwise protocol to treat chronic, incapacitating pain in patients with ADPKD [11,12,13]. This treatment protocol includes several steps to achieve acceptable pain control, among which are nonpharmacological, pharmacological, minimally invasive and invasive treatments. Options for minimally invasive treatments are nerve blocks (celiac plexus blocks and splanchnic nerve blocks) and cyst aspirations/fenestrations, while invasive treatment includes nephrectomy. In a study published in 2023 in which we investigated 101 patients, we found that this treatment protocol led to substantial and sustained pain relief in the majority of patients [11].

To our knowledge, there is no stepwise approach for the management of chronic kidney-related pain in non-ADPKD patients. Given the favorable efficacy, we assessed in this case series whether our treatment protocol for ADPKD-related pain can also provide sufficient pain relief in non-ADPKD patients with chronic, refractory, incapacitating pain.

## 2. Methods

### 2.1. Study Population

From January 2014 until April 2022, patients with chronic, severe kidney-related pain were screened at the pain clinic of our Expertise Center for Polycystic Kidney Diseases at the University Medical Center Groningen (UMCG), the Netherlands. Some patients referred themselves, while others were referred by their treating physician from all over the Netherlands. All treated patients were included in this analysis. Written informed consent was obtained from all included subjects. Participants were given time to consider participation and the option to ask questions about the study face to face or by telephone. The signed informed consent forms were stored at the UMCG. Indications for referral were pain with a pain score on a visual analog scale (VAS) ≥50 out of 100 that were likely to be kidney-related, lasting ≥3 months, were incapacitating (negatively affecting physical and/or social life according to self-assessment) and had insufficient response to previous pain treatments. This case series was approved by the Central Ethics Review Board of the UMCG (ID 19369) and conducted in adherence with the International Conference on Harmonization Good Clinical Practice guidelines.

### 2.2. Adapted Treatment Protocol

**Figure 1:**
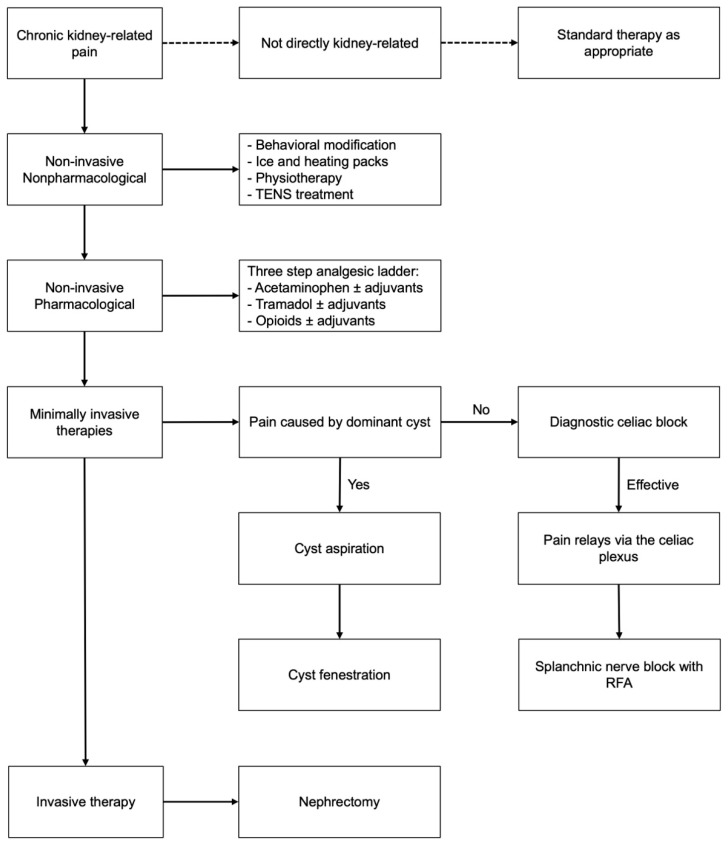
Adapted treatment protocol for chronic refractory kidney-related pain.

Patients were analyzed and treated according to a protocol originally developed to manage ADPKD-related pain, which has been described extensively before [11,12,13]. This protocol was adapted for kidney-related pain specifically and no longer includes treatment of liver-related pain (Figure 1). After referral, patients had an intake with a nephrologist or urologist, depending on the cause of pain, as well as with a pain specialist. Non-kidney-related pain was excluded by performing a history and laboratory assessments, as well as magnetic resonance imaging (MRI), computed tomography (CT) scan and radioisotope renography when deemed necessary. The findings were then discussed in a multidisciplinary meeting with a nephrologist, urologist, radiologist, pain specialist and, when indicated, a gastroenterologist, and an appropriate treatment was chosen to be discussed with the patient.

As a first step, nonpharmacological therapies (including transcutaneous electrical nerve stimulation (TENS)) and analgesics were prescribed or optimized. When needed, adjuvant medication such as sleep medication or specific antidepressants were prescribed. During the pre-treatment work-up, potential causes for kidney-related pain were investigated as a target for treatment. In case of a residual stone load, surgery was performed to achieve sufficient stone removal. If nonpharmacological therapies and analgesics did not result in pain relief and a parapelvic/simple cyst was thought to be the cause of pain, a cyst aspiration without sclerosant was performed. If successful, this was followed by (robot-assisted) laparoscopic fenestration upon pain recurrence. For patients dependent on kidney replacement therapy (KRT) and fit for surgery, native nephrectomy was the preferred option.

If there were no cysts and the patient was not KRT-dependent, nerve blocks were indicated. First, a diagnostic short-acting retrocrural celiac plexus block was performed on the most painful side under local anesthetic. If the VAS score decreased by approximately 20%, the procedure was considered successful. In the case of the recurrence of pain, these patients were scheduled for a splanchnic block with radiofrequency ablation (RFA), of which the effect is expected to be longer. A diagnostic celiac plexus block or RFA of the major splanchnic nerves takes approximately 30 min. Post-procedure, patients are closely monitored for 2–4 h until discharge. More specific information on these nerve blocks is described elsewhere [11,13].

If pain relief was achieved after the splanchnic RFA block but recurred after some time, the splanchnic block was repeated. If no substantial pain relief was observed after the diagnostic celiac plexus or splanchnic nerve block, a nephrectomy was considered. Depending on the cause of pain, additional treatment was initiated before or simultaneously with pain therapy (e.g., kidney stone prevention).

### 2.3. Data Collection

The primary outcome was the effect of treatment on pain and quality of life, as expressed by VAS scores (0–100), defined daily dose of pain medication, Short Form Health Survey 36 (SF-36) component scores and Patient Health Questionnaire (PHQ-9) scores [14,15]. Considering the minimal clinically important difference of the VAS score [16], a reduction in VAS score of >20% was considered substantial pain relief. The SF-36 is a validated health-related quality of life questionnaire containing 36 items, which can be translated into a physical component score (PCS) and mental component score (MCS), both ranging from 0 to 100, with a lower score indicating a worse quality of life [14]. The PHQ-9 contains nine items concerning depression, with each item being rated on a four-point Likert scale, resulting in a score ranging between 0 and 27, with a higher score indicating a worse quality of life [15]. These outcomes were evaluated and compared at three time points: before treatment, at short-term follow-up (6 weeks post-treatment) and at long-term follow-up. These data were obtained through the assessment of electronic patient files and a retrospective questionnaire in which patients had to complete information concerning their pain and quality of life at the three time points. The questionnaire is included in the Supplementary Materials. In addition, adverse events after treatment were assessed in patient records.

### 2.4. Sample Size Calculation

The sample size calculation was based on a paired t-test. The previous study with 101 patients with ADPKD showed a mean VAS score of 60 ± 30 and found a reduction of 66% to 20. We expected to find a similar change in the VAS score of 40. We applied 80% power and a two-tailed alpha of 0.05. Based on these parameters, we found a required sample size of 7 participants.

### 2.5. Statistical Analysis

Continuous data are presented as median [interquartile rage (IQR)] and categorical data as percentages. A comparison of outcomes between time points was performed using a paired Wilcoxon signed rank test. The PCS and MCS were calculated using the method described by Ware et al. [17]. A two-sided *p*-value < 0.05 was considered statistically significant. Statistical analyses and visualization were performed using R version 4.3.2 (Vienna, Austria).

## 3. Results

**Figure 2:**
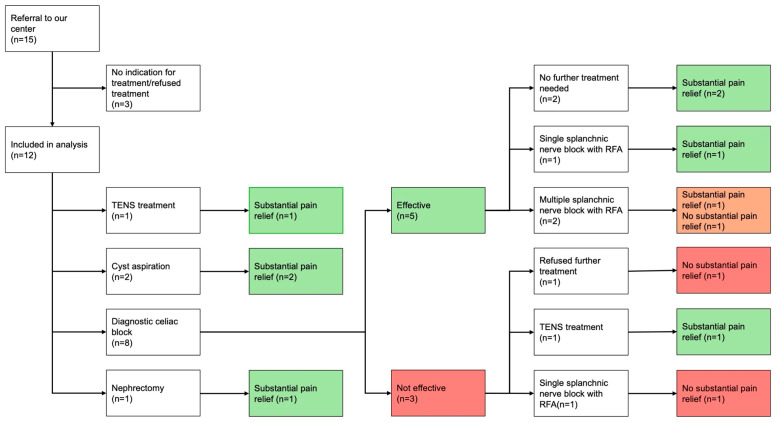
Flowchart of patients treated for kidney-related pain.

Fifteen patients were referred to our center in the context of the clinical treatment protocol. Of these patients, 3 did not receive treatment because there was no indication for it (*n* = 1, long-term antibiotic treatment was deemed more appropriate) or declined the treatment that was offered (*n* = 1, cyst aspiration, and *n* = 1, celiac block, both declined because pain was not severe enough to warrant treatment), leaving 12 patients for the analysis (Figure 2). Of the treated patients, one received TENS treatment as initial treatment. In terms of (minimally) invasive treatment, two patients received a cyst aspiration, eight patients received a diagnostic celiac block and one patient underwent nephrectomy. In the eight patients that received a diagnostic celiac block, six needed further treatment, which was refused by one patient (wished to focus on another medical issue), whereas the remaining five received TENS treatment (*n* = 1), a single splanchnic nerve block with RFA (*n* = 2) or multiple treatments with a splanchnic nerve block with RFA (*n* = 2).

### 3.1. Patient and Pain Characteristics

The patients that were referred to our pain clinic were more often female (*n* = 8, 67%) and had a median age of 50 [IQR: 36–59] years (Table 1). The median estimated glomerular filtration rate (eGFR) was 80 [IQR: 71–89] mL/min/1.73 m^2^, and none of the patients were kidney-replacement-therapy-dependent. Detailed characteristics of individual patients are reported in Supplementary Table S1.

The assumed predisposing causes of chronic pain were a history of kidney stones (*n* = 6, 50%), an anatomic abnormality of the kidney (*n* = 3, 25%), a history of upper urinary tract infection (*n* = 1, 8%), previous kidney surgery (*n* = 1, 8%), loin pain hematuria syndrome (*n* = 1, 8%), previous radiotherapy for prostate cancer (*n* = 1, 8%) or a combination of these events (Table 2). The median duration of pain before consultation was 1.9 [IQR: 1.0–4.7] years, and the average VAS score was 70 [IQR: 48–90] out of 100. Pain was located on the right side of the abdomen in eight patients, on the left side in two patients and on both sides in two patients. Before the first consultation, all patients had been treated by analgesics and nonpharmacological treatments (such as physiotherapy), whereas at the time of consultation most still received (a combination of) non-opioids (*n* = 8, 75%) and opioids (*n* = 8, 75%), with one patient who had undergone an epidural nerve block previously. In addition to kidney-related pain, one patient had chronic musculoskeletal pain syndrome of both legs, and one patient had tension headaches. Pain characteristics of separate patients are reported in Supplementary Table S2.

### 3.2. Protocolized Treatment

**Figure 3:**
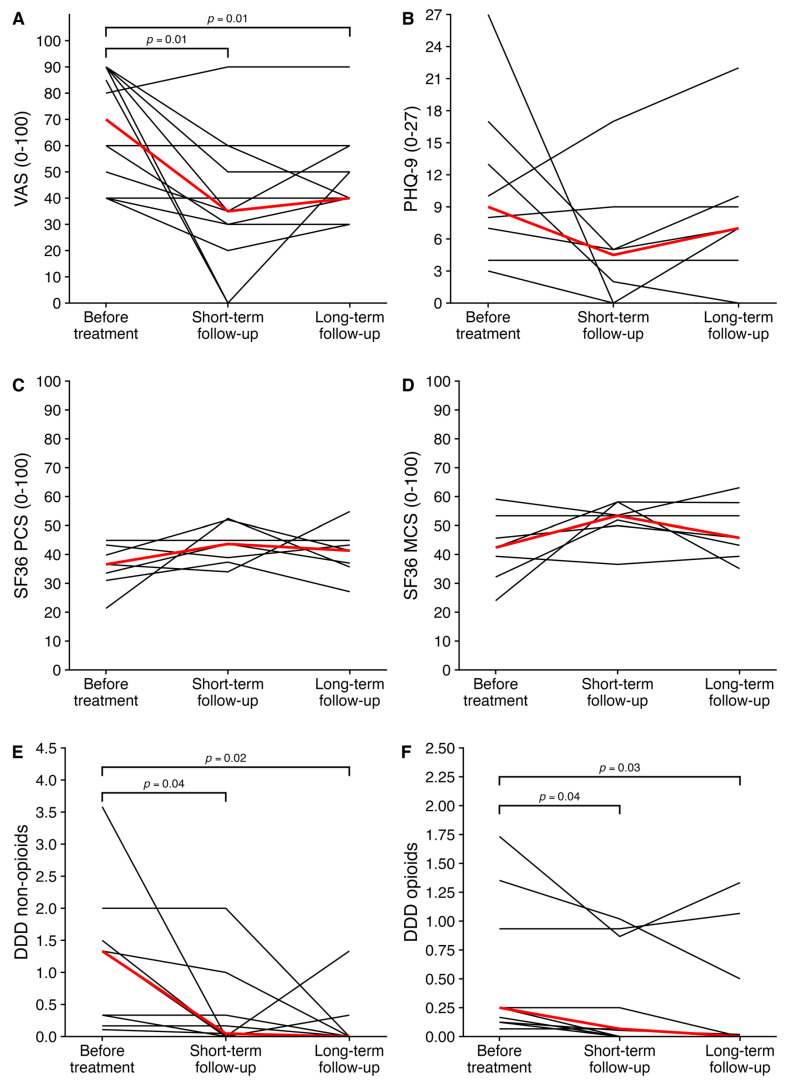
Changes in outcome variables. Statistically significant differences between time points are indicated at the top of the graphs. The red line indicates the median change in outcomes. (**A**) VAS score. A higher score indicates higher pain experience. (**B**) PHQ-9. A higher score indicates a worse quality of life. (**C**) SF36 PCS. A higher score indicates a better quality of life. (**D**) SF36 MCS. A higher score indicates a better quality of life. (**E**) DDD non-opioids. (**F**) DDD opioids. Abbreviations: DDD, defined daily dose; MCS, mental component score; PCS, physical component score; PHQ, patient health questionnaire; SF36, short form 36; VAS, visual analog scale.

Of the total patients, 75% (*n* = 9) had substantial pain relief at short-term follow-up after the last intervention (Table 3). Compared to before the treatment, the median VAS score was decreased by 35 points at short-term follow-up after the last given treatment, from 70 [IQR: 48–90] to 35 [IQR: 28–53] (*p* = 0.01), indicating pain relief of 50% (Figure 3). In addition to a reduction in pain scores, in the subjects that received opioids, the median defined daily dose was significantly reduced at short-term follow-up (from 0.25 [IQR: 0.12–0.93] to 0.07 [IQR: 0–0.87], *p* = 0.04). A reduction was also found in patients who were treated with non-opioids before treatment (from 1.33 [IQR: 0.33–1.50] to 0.05 [IQR: 0–0.33] at short-term follow-up, *p* = 0.04). Assessment of quality of life suggested an improvement in the PCS, MCS and PHQ-9 scores at short-term follow-up, although this did not reach formal statistical significance (PCS: 36.6 [32.3–41.5] to 43.6 [38.1–48.4], *p* = 0.1; MCS: 42.4 [35.8–49.5] to 53.4 [50.9–55.8], *p* = 0.2; PHQ-9: 9.0 [6.3–14.0] to 4.5 [1.5–6.0], *p* = 0.2).

### 3.3. Long-Term Follow-Up

The median follow-up time was 3.8 [IQR: 2.5–4.4] years after the last treatment. At long-term follow-up, all patients who had pain relief at short-term follow-up still experienced pain relief (Table 3). At that time point, the median VAS score remained decreased at 40 [IQR: 38–53] (*p* = 0.01). The defined daily dose of opioids was also still significantly reduced at long-term follow-up (0 [IQR: 0–0.50], *p* = 0.03), as well as the defined daily dose of non-opioids (0 [IQR: 0–0.02], *p* = 0.02) in those patients who received this medication before therapy. Similarly, during long-term follow-up, an improvement in quality of life was suggested (PCS 36.6 [32.3–41.5] to 41.3 [36.3–44.1], *p* = 0.3; MCS 42.4 [35.8–49.5] to 45.7 [41.3–55.6], *p* = 0.1; PHQ-9 (9.0 [6.3–14.0] to 7.0 [3.0–9.3], *p* = 0.3). Detailed results of the treatment protocol in individual patients are reported in Supplementary Table S3. Of note, the patient who underwent a nephrectomy was completely pain-free and did not require any analgesics after nephrectomy. Three patients had no response on treatment but refused further treatment.

### 3.4. Adverse Events

There were no adverse events during the various interventions, one adverse event immediately after the procedure (orthostatic hypotension after a celiac block) and one adverse even during the first eight weeks post-procedure (fatigue after nephrectomy).

### 3.5. Subgroup Analysis

A subgroup analysis was performed based on the assumed cause of pain, with two subgroups being analyzed (assumed cause of pain: kidney stones (*n* = 6) and other causes of pain (*n* = 6)). None of the changes in pain, pain medication dosages or quality of life were statistically significant (Supplementary Table S4). Descriptively, patients who had chronic pain due to kidney stones had a short- and long-term reduction in VAS (73 [53–89] to 38 [9–48] and 45 [40–50], respectively). These patients also had a decrease in the defined daily dose of non-opioids (1.50 [1.33–2.00] to 0 [0–1.00] short-term and 0 [0–0] long-term) and opioids (0.59 [0.16–1.25] to 0.47 [0.02–0.92] short-term and 0.25 [0–0.93] long-term). In addition, quality-of-life scores seemed to improve, although these changes were not statistically significant. These results were overall comparable to those in patients with another assumed cause of pain, and with the limitations of statistical power, no large differences in response were found between these two groups.

## 4. Discussion

In this prospective case series, we found that our multidisciplinary, step-wise protocol that was originally developed to treat chronic, incapacitating kidney-related pain in patients with ADPKD was also effective in reducing pain and opioid use in well-selected patients with chronic, incapacitating kidney-related pain in non-ADPKD patients. These benefits were observed both at short- and long-term follow-up.

The literature on the treatment of non-ADPKD kidney-related pain is limited. Small studies in patients with loin pain hematuria syndrome are noted, but likely due to the low prevalence of this syndrome (approximately 0.012% in the general population) together with the often mild symptomatology, no large cohorts have been described [18]. In addition, no data has been reported in patients suffering from chronic pain due to sensitization, which can occur after an acute pain episode. This causes the central nervous system to increase responsiveness to normally innocent sensory stimuli, resulting in pain [19]. What is known is that pain generally has a negative impact on quality of life. Also in the current case series, quality of life, assessed as the PCS (35.4) and MCS (43.9) of the SF-36 questionnaire, was impaired compared to the PCS (53.6) and MCS (50.4) in the general population [20]. Similar results were found for the PHQ-9 score, as the PHQ-9 score before treatment in this case series was 9.0 and in a general population cohort was 2.9 [21]. This impaired quality of life is in line with our findings in ADPKD patients with chronic kidney-related pain, indicating that there is a need for effective treatments of kidney-related chronic pain.

Several treatment options for non-ADPKD kidney-related pain have been described in the literature, such as local infusions with anesthetics, intra-ureteric infusion of capsaicin, lumbar sympathetic chain neuromodulation, nerve blocks, surgical and catheter-based renal denervation, nephrectomy and autotransplantation [2,18,22,23,24,25,26,27]. These interventions have different success rates in treating kidney-related pain. Local infusions with the anesthetic bupivacaine or opioids have a positive response rate between 25 and 100% but were effective in most cases for only a short period [18]. Intra-ureteric infusion of capsaicin has also been described to be effective for a short period but may have a nephrotoxic effect, resulting in kidney function decline [28]. Lumbar sympathetic chain neuromodulation may also be a potential option. Positive results with respect to pain relief were described in a case series including four patients published in 2009, but follow-up time was short, and up to now only one other case report has been published describing this method for kidney-related pain [23,29]. Another treatment used in patients with kidney-related pain is renal denervation. This can be performed surgically or by a catheter-based technique. Of these methods, catheter-based renal denervation has been described to be slightly more effective, although there are no studies directly comparing these techniques and all studies contained relatively small populations. Overall, the success rate of renal denervation has been described to be between 15 and 100% in a total of 103 patients included in five studies [2,18,24,25,30]. Last-resort options are performing a nephrectomy or autotransplantation [26,27]. By performing a native nephrectomy and transplanting the kidney into the iliac fossa of the same patient, the renal nerves are transected, preventing pain signals from reaching the central nervous system. There have been multiple publications describing this method, of which one utilizes a robot-assisted approach [27,30]. In a review including in total 220 patients, success rates were high (71–90%) [30], but there was also an intermediate risk for surgical and long-term complications, such as an afunctional kidney transplant or intestinal perforation. In our center, nephrectomy was chosen as the treatment of choice compared to autotransplantation.

Our treatment protocol had a success rate of ~70% in a small group of well-selected patients and included mostly non- to minimally invasive treatments. Nerve blocks are an important step in a percentage of patients treated with this protocol. These blocks may be especially effective in treating chronic pain caused by sensitization after an episode of acute pain, as indicated by two of our patients, in whom a diagnostic celiac block was effective in providing sustained pain relief without the need for a long-lasting splanchnic nerve block. Such a diagnostic celiac block is performed using the short-acting anesthetic bupivacaine and should only be effective for a short time period. However, this block may be effective for a longer period through interruption of the vicious pain cycle caused by sensitization [3,31]. Sensitization is a complex phenomenon in which peripheral inputs are thought to be one of the factors maintaining the condition [32]. By removing this peripheral input through a nerve block, nociceptive neurons with increased responsiveness may reset, therefore reducing the pain perceived due to this phenomenon. Hypothetically, this treatment may also be effective in patients in whom sensitization is the result of an acute pain episode in other organs or tissue. However, whether this is indeed true needs additional study.

In addition to the symptomatic treatment of pain, the prevention of new kidney stones is a cornerstone in the treatment of stone-forming patients. Patients should be advised to increase their fluid intake to >2500–3000 mL per day, lower daily salt intake to <4–5 g and limit animal protein intake [33]. Further, the residual stone load should be low after kidney stone treatment, as recurrence is high when there are residual stone fragments, especially those >4 mm [34].

In one of our patients, nephrectomy was performed with an excellent result with respect to pain relief. It should be noted, however, that nephrectomy is a last-resort option with an intermediate risk for complications and especially a negative effect on kidney function. Therefore, nephrectomy should only be considered in well-selected patients after discussion of the risks of nephrectomy weighed against the possible results in a process of shared decision making.

We found an improvement in all quality-of-life scores at short-term as well as at long-term follow-up, although these changes were not statistically significant. This is likely caused by our small study population [35]. Future studies with larger populations need to be performed to investigate the effect of this treatment protocol on quality of life.

This case series has limitations, the main one being the relatively small population, which decreases statistical power and increases the chance of a Type 2 statistical error. Selection and recall bias were avoided by including and describing all patients treated at our center using this treatment protocol and by prospectively collecting per-protocol pain scores. The use of the structured follow-up is, therefore, the main strength. A further limitation is that we were unable to compare the results of our protocol with a matched control group, as we included a very specific group of patients, which is difficult to match properly. Since pain in our patients was chronic, stable and not responding to various other pain-reducing strategies, and pain improved shortly after the intervention, it is unlikely that this improvement was due to a spontaneous remission.

## 5. Conclusions

In conclusion, this prospective case series showed that our multidisciplinary, stepwise treatment protocol is effective in achieving substantial and sustained pain relief in the majority of non-ADPKD patients with chronic, incapacitating kidney-related pain. We propose, therefore, that this protocol can be used in such patients to improve their quality of life. Because of the heterogeneity of this patient group, a multidisciplinary patient selection is of utmost importance.

## Figures and Tables

**Table 1:** Patient characteristics.

	*n* = 12
Age (years)	50 [36–59]
Female sex, *n* (%)	8 (67)
Height (cm)	174 [167–180]
Weight (kg)	83 [71–93]
Body mass index (kg/m^2^)	27 [25–33]
History of	
-Kidney stones, *n* (%)	8 (68)
-Urinary tract infection, *n* (%)	4 (33)
-Upper urinary tract infection, *n* (%)	3 (25)
-Episodes of macroscopic hematuria, *n* (%)	4 (33)
-Kidney surgery, *n* (%)	2 (17)
Systolic blood pressure (mmHg)	130 [120–143]
Diastolic blood pressure (mmHg)	83 [74–89]
Use of blood pressure lowering drugs, *n* (%)	6 (50)
eGFR (mL/min/1.73 m^2^)	80 [71–89]
Short Form-36 Score	
-Physical component score (0–100)	36.6 [32.3–41.5]
-Mental component score (0–100)	42.4 [35.8–49.5]
PHQ-9 score (0–27)	9.0 [6.3–14.0]

Continuous values are reported as median [IQR]. Abbreviations are as follows: eGFR, estimated glomerular filtration rate; PHQ, patient health questionnaire.

**Table 2:** Pain characteristics.

	*n* = 12
Duration of pain (years)	1.9 [1.0–4.7]
Pain severity last 4 weeks	
-Minimum VAS score (0–100)	55 [38–63]
-Maximum VAS score (0–100)	80 [75–91]
-Average VAS score (0–100)	70 [48–90]
Assumed cause of chronic pain *	
-Kidney stones, *n* (%)	6 (50)
-Anatomic abnormality of the kidney, *n* (%)	3 (25)
-Upper urinary tract infection, *n* (%)	1 (8)
-Kidney surgery, *n* (%)	1 (8)
-Loin pain hematuria syndrome, *n* (%)	1 (8)
-Previous radiotherapy, *n* (%)	1 (8)
Patient reported location as **	
-Left kidney, *n* (%)	4 (33)
--*Ventral side*	3 (25)
--*Dorsal side*	4 (33)
-Right kidney, *n* (%)	10 (83)
--*Ventral side*	8 (67)
--*Dorsal side*	9 (75)
Management of pain	
-Nonpharmacological therapies, *n* (%)	
--*Physiotherapy*	4 (33)
--*Cognitive behavioral therapy*	2 (14)
-Pharmacological therapies, *n* (%)	
--*Acetaminophen*	8 (67)
--*NSAID*	5 (42)
--*Sleep medication*	3 (25)
--*Low-dose opioids*	5 (42)
--*High-dose opioids*	5 (42)
-Previous invasive pain therapies, *n* (%)	
--*Nerve block*	2 (17)

Continuous values are reported as median [IQR]. Abbreviations are as follows: VAS score, visual analogue scale score; NSAIDs, non-steroidal anti-inflammatory drugs. *, Both an upper urinary tract infection and kidney surgery were the assumed cause of pain in one patient, and both a kidney abnormality and an upper urinary tract infection in one other patient. **, Patients could have pain in both kidneys and on both the ventral and dorsal side.

**Table 3:** Overall results of last pain treatment, short-term follow-up and long-term follow-up.

	Before Treatment	Short-Term Follow-Up		Long-Term Follow-Up	
		Characteristics	*p*-Value	Characteristics	*p*-Value
Positive effect last intervention (%)	-	75	-	75	-
VAS score (0–100)	70 [48–90]	35 [28–53]	0.01	40 [38–53]	0.01
Defined daily dose non-opioids *	1.33 [0.33–1.50]	0.05 [0–0.33]	0.04	0 [0–0.02]	0.02
Defined daily dose opioids *	0.25 [0.12–0.93]	0.07 [0–0.87]	0.04	0 [0–0.50]	0.03
Physical component score (0–100)	36.6 [32.3–41.5]	43.6 [38.1–48.4]	0.1	41.3 [36.3–44.1]	0.3
Mental component score (0–100)	42.4 [35.8–49.5]	53.4 [50.9–55.8]	0.2	45.7 [41.3–55.6]	0.1
PHQ-9 score (0–27)	9.0 [6.3–14.0]	4.5 [1.5–6.0]	0.2	7.0 [3.0–9.3]	0.3

Continuous values are reported as median [IQR]. Abbreviations: *n*, number; VAS score, visual analogue scale score; PHQ, patient health questionnaire. * Defined daily dose of non-opioids and opioids was analyzed in patients who used these medications before treatment (*n* = 9 and *n* = 9, respectively).

## Data Availability

All data are included in the manuscript and/or Supplementary Materials.

## Supplementary Materials

The following supporting information can be downloaded at https://www.mdpi.com/article/10.3390/jcm14165623/s1. Table S1: Characteristics of individual patients. Table S2: Pain characteristics of individual patients. Table S3: Results last pain treatment in individual patients at short-term follow-up and long-term follow-up. Table S4: Subgroup analysis based on the assumed cause of chronic pain. Questionnaire follow-up pain treatment
