# Supplementary material for: Results of a Multidisciplinary Stepwise Protocol to Treat Chronic Refractory Kidney-Related Pain
Source: J Clin Med. 2025 Aug 8;14(16):5623. doi: 10.3390/jcm14165623 (PMC12386564; doi:10.3390/jcm14165623)
Supplement: Supplementary file 1 [file jcm-14-05623-s001.zip › jcm-3703247-supplementary.pdf]

Table of contents

- **Table S1.** Characteristics of individual patients
- **Table S2**. Pain characteristics of individual patients
- **Table S3**. Results last pain treatment in individual patients at short-term follow-up and long-term follow-up
- **Table S4**. Subgroup analysis based on the assumed cause of chronic pain
- **Questionnaire follow-up pain treatment**

Table S1. Characteristics of individual patients

S	Age/Sex	Height (cm)	Weight (kg)	History of UTI	History of Upper UTI	History of Episodes of macr. hematuria	History of Kidney stones	History of Kidney surgery	Blood pressure (mmHg)	eGFR (mL/min/1.73m^2)
1	59/Female	170	92.2	Y	Y	Y	Y	N	108/57	66
2	45/Female	178	70.8	Y	Y	Y	Y	N	144/84	80
3	47/Male	174	77.0	N	N	Y	Y	N	-	-
4	22/Female	184	83.0	N	N	N	N	N	128/78	61
5	57/Female	168	70.0	N	N	N	Y	Y	118/82	77
6	60/Female	165	84.0	N	N	N	Y	Y	127/73	51
7	53/Male	184	130.0	N	N	N	Y	N	-	88
8	38/Male	181	118.0	N	N	N	Y	N	113/64	76
9	31/Female	165	94.5	N	N	Y	Y	N	141/90	110
10	77/Female	-	61.0	Y	N	N	N	N	143/89	82
11	29/Female	165	55.0	Y	Y	N	N	N	132/87	107
12	63/Male	176	83.0	N	N	N	N	N	163/106	89

Abbreviations: eGFR, estimated glomerular filtration rate; KRT, kidney replacement therapy; macr, macroscopic; N, no; S, subject; UTI, urinary tract infection; Y, yes.

Table S2. Pain characteristics of individual patients

S	Duration pain (years)	Assumed pain cause	VAS score	Pain location		Pain related to				Therapies			
				Left vs. right	Ventral vs. dorsal	Intake	Mict.	Def.	Physical activity	Non-opioids use	Opioids use	Sleep med. use	Previous invasive therapies
1	1.05	Kidney stones	50	Right	Dorsal	N	N	N	Y	Y	Y	N	N
2	0.77	Kidney stones	85	Right	Both	N	N	N	N	Y	Y	N	N
3	2.29	Anatomic abnormality of the kidney	40	Right	Both	N	N	N	N	Y	Y	N	N
4	6.15	Loin pain hematuria syndrome	90	Left	Both	N	N	N	Y	Y	Y	N	N
5	7.15	Kidney surgery	60	Right	Both	N	Y	N	Y	Y	N	N	N
6	13.88	Kidney stones	90	Both	Both	N	N	N	N	Y	Y	N	N
7	1.51	Kidney stones	90	Right	Ventral	N	N	N	N	Y	Y	N	Y
8	4.27	Kidney stones	60	Right	Both	N	N	N	N	N	Y	N	N
9	0.46	Kidney stones	40	Right	Dorsal	N	N	N	Y	Y	Y	Y	N
10	1.07	Anatomic abnormality of the kidney	40	Left	Dorsal	N	N	N	Y	Y	Y	N	Y
11	2.69	Anatomic abnormality of the kidney + upper urinary tract infection	90	Both	Both	N	N	N	N	N	N	Y	N
12	0.42	Previous radiation therapy	80	Right	Both	N	N	N	Y	N	N	Y	N

Abbreviations: def, defecation; max, maximum; mict, micturition; min, minimum; N, no; S, subject; VAS score, visual analogue scale score; Y, yes.

Table S3. Results last pain treatment in individual patients at short-term follow-up and long-term follow-up

S	Last pain treatment	VAS score			Defined Daily Dose non-opioids			Defined Daily Dose opioids.		
		Before treatment	Short-term follow-up	Long-term follow-up	Before treatment	Short-term follow-up	Long-term follow-up	Before treatment	Short-term follow-up	Long-term follow-up
1	Diagnostic celiac block	50	35	40	2.00	2.00	0	0.07	0.07	0
2	Nephrectomy	85	0	0	3.58	0	0	0.25	0	0
3	Cyst aspiration	40	20	30	0.17	0.17	0	0.17	0	0
4	TENS treatment	90	35	60	0.11	0.05	0.02	0.13	0.05	0.02
5	Splanchnic nerve block with RFA	60	30	40	0.33	0	0.33	0	0	0
6	Diagnostic celiac block	90	0	50	1.33	0	1.33	0.12	0	0
7	Splanchnic nerve block with RFA	90	50	50	1.33	1.00	0	1.35	1.02	0.50
8	Splanchnic nerve block with RFA	60	60	60	0	0	0	1.73	0.87	1.33
9	Splanchnic nerve block with RFA	40	40	40	1.50	0	0	0.93	0.93	1.07
10	Cyst aspiration	40	30	30	0.33	0.33	0	0.25	0.25	0
11	TENS treatment	90	60	40	0	0	0	0	0	0
12	Diagnostic celiac block	80	90	90	0	0	0	0	0	0

Abbreviations: PHQ, patient health questionnaire; RFA, radiofrequency ablation; S, subject; TENS, transcutaneous electrical nerve stimulation; VAS score, visual analogue scale score;

Table S4. Subgroup analysis based on the assumed cause of chronic pain*

**Kidney stones (n=6)**	**Before treatment**	**Short-term follow-up**	**Long-term follow-up**	**P-value** before vs. short	**P-value** before vs. long
Positive effect last intervention (%)	-	67	67	-	-
VAS score (0-100)	73 [53 – 89]	38 [9 – 48]	45 [40 – 50]	0.1	0.1
Defined Daily Dose non-opioids**	1.50 [1.33 – 2.00]	0 [0 – 1.00]	0 [0 – 0]	0.1	0.1
Defined Daily Dose opioids**	0.59 [0.16 – 1.25]	0.47 [0.02 – 0.92]	0.25 [0 – 0.93]	0.1	0.2
Physical Component Score (0-100)	39.9 [32.8 – 43.7]	41.9 [37.7 – 46.8]	44.1 [41.4 – 47.4]	0.9	0.4
Mental Component Score (0-100)	40.9 [35.5 – 45.1]	55.7 [49.2 – 58.1]	46.4 [38.3 – 54.5]	0.4	0.4
PHQ-9 score (0-27)	10.0 [4.0 – 13.0]	2.0 [0 – 4.0]	4.0 [0 – 7.0]	0.4	0.4

*Continues values are reported as median [IQR]. Abbreviations: n, number; VAS score, visual analogue scale score; PHQ, patient health questionnaire. * Subgroup analysis was performed for kidney stones as the assumed cause of pain. ** Defined Daily Dose of non-opioids and opioids were analyzed in patients who used these medications before treatment (kidney stones: n=5 and n=6). *** Quality of life scores were not analyzed because two patients did not complete the questionnaires.*

Vragenlijst patiënt:
Protocollaire behandeling chronische flankpijn

Toelichting: Met behulp van deze vragenlijst hopen we een beeld te krijgen van uw pijnklachten en het effect van behandeling. Deze vragenlijst bestaat uit drie onderdelen, genummerd 1 tot en met 3. Deze delen bevatten vragen over uw algemene gezondheid en specifieke klachten op een bepaald moment in uw leven, ten opzichte van de pijn behandeling die u destijds bent ondergaan. Op deze manier kunnen wij de klachten op deze momenten met elkaar vergelijken. Deel 1 vraagt naar uw gezondheidstoestand op dit moment, deel 2 naar uw gezondheidstoestand voor uw pijnbehandeling en deel 3 naar uw gezondheidstoestand 6 weken na uw pijnbehandeling.

Zou u een kruis willen zetten in het hokje dat voor u het best het antwoord weergeeft? Vergeet u niet om alle vragen in te vullen. U kunt deze vragenlijst terugsturen in de bijgevoegde envelop. Mocht u aanvullende vragen hebben, neem dan gerust contact met ons op. Alvast heel hartelijk dank.

Beantwoord deze vragen a.u.b. zo eerlijk en zo duidelijk mogelijk. Beantwoord elke vraag door één hokje te markeren. Als u een vraag niet precies kunt beantwoorden, geef dan het best mogelijke antwoord.

Overzicht

Deel 1: Gezondheid <u>op dit moment</u>

Deel 1A – Pijnklachten en medicatie 2
Deel 1B – Patiënt geestelijke gezondheid - Patient Health Questionnaire (PHQ-9) 3
Deel 1C – Algehele gezondheid en kwaliteit van leven (SF-36) 4
Deel 1D – Uw nierziekte 7

Deel 2: Gezondheid <u>voor de pijnbehandeling</u>

Deel 2A – Pijnklachten en medicatie 10
Deel 2B – Patiënt geestelijke gezondheid - Patient Health Questionnaire (PHQ-9) 11
Deel 2C – Algehele gezondheid en kwaliteit van leven (SF-36) 12
Deel 2D – Uw nierziekte 14

Deel 3: Gezondheid <u>6 weken na pijnbehandeling</u>

Deel 3A – Pijnklachten en medicatie 18
Deel 3B – Patiënt geestelijke gezondheid - Patient Health Questionnaire (PHQ-9) 19
Deel 3C – Algehele gezondheid en kwaliteit van leven (SF-36) 20
Deel 3D – Uw nierziekte 22

Algemene gegevens

1.	Naam patiënt	__________________________
2.	Geboortedatum	______/______/___________
3.	Datum afname vragenlijst	______/______/___________

Deel 1: Gezondheid op dit moment

Deel 1A: Pijnklachten

1.	Wel eens nierpijn op dit moment?	1 ☐ Ja	0 ☐ Nee
1b.	Zo ja, waar?	1 ☐ Links	0 ☐ Rechts
1c.	Zo ja, frequentie nierpijn per dag?		__________________keer
1d.	Zo ja, frequentie nierpijn per week?		__________________keer
1e.	Zo ja, ooit opgenomen vanwege nierpijn?	1 ☐ Ja	0 ☐ Nee
1f.	Gemiddelde pijn score nieren (VAS score) gemiddeld over de afgelopen week? (0 – 100)		_________________score
1g.	Maximale pijn score nieren (VAS score) over de afgelopen week? (0 – 100)		_________________score
1h.	Minimale pijn score nieren (VAS score) gemiddeld over de afgelopen week? (0 – 100)		_________________score
1i.	Pijn score nieren (VAS score) op dit moment (0 – 100)		_________________score

Medicatie

1.	Gebruikt u op dit moment pijnmedicatie?	1 ☐ Ja 0 ☐ Nee
1b.	Zo ja, welke?	1 ☐ Paracetamol
		2 ☐ NSAID's (o.a. ibuprofen, naproxen, diclofenac)
		3 ☐ Morfinepreparaten
		4 ☐ Anders, namelijk______________________
2.	Graag alle huidige medicatie met doseringen noteren:	____
		____
		____
		____

Deel 1B: Patiënt geestelijke gezondheid - Patient Health Questionnaire (PHQ-9)

*Hoe vaak hebt u in de **afgelopen 2 weken** last gehad van één of meer van de volgende problemen?*	Helemaal niet	Verschei-dene dagen	Meer dan de helft van de dagen	Bijna elke dag
1. Weinig interesse of plezier om dingen te doen	0 ☐	1 ☐	2 ☐	3 ☐
2. Zich neerslachtig, gedeprimeerd of hopeloos voelen	0 ☐	1 ☐	2 ☐	3 ☐
3. Moeilijk inslapen, moeilijk doorslapen of teveel slapen	0 ☐	1 ☐	2 ☐	3 ☐
4. Zich moe voelen of gebrek aan energie hebben	0 ☐	1 ☐	2 ☐	3 ☐
5. Weinig eetlust of overmatig eten	0 ☐	1 ☐	2 ☐	3 ☐

6.	Een slecht gevoel hebben over uzelf – of het gevoel hebben dat u een mislukking bent of het gevoel dat u zichzelf of uw familie teleurgesteld hebt	0 ☐	1 ☐	2 ☐	3 ☐
7.	Problemen om u te concentreren, bijvoorbeeld om de krant te lezen of om tv te kijken	0 ☐	1 ☐	2 ☐	3 ☐
8.	Zo traag bewegen of zo langzaam spreken dat andere mensen dit opgemerkt kunnen hebben? Of het tegenovergestelde, zo zenuwachtig of rusteloos zijn dat u veel meer bewoog dan gebruikelijk	0 ☐	1 ☐	2 ☐	3 ☐
9.	De gedachte dat u beter dood zou kunnen zijn of de gedachte uzelf op een bepaalde manier pijn te doen	0 ☐	1 ☐	2 ☐	3 ☐

For office coding ___0___ + ______ + ______ + ______

Total score: ________

10. Als u **enig** probleem hebt aangekruist, hoe **moeilijk** maakten deze problemen het dan voor u om uw werk of uw taken in en om het huis te doen, of om met andere mensen om te gaan?

1 ☐ Helemaal niet moeilijk 2 ☐ Enigszins moeilijk 3 ☐ Erg moeilijk 4 ☐ Extreem moeilijk

Deel 1C: Algehele gezondheid en kwaliteit van leven (SF-36)

Uw gezondheid

1.	Hoe zou u over het algemeen uw gezondheid noemen?	1 ☐ Uitstekend<br>2 ☐ Zeer goed<br>3 ☐ Goed<br>4 ☐ Matig<br>5 ☐ Slecht
2.	Hoe beoordeelt u nu uw gezondheid in het algemeen, vergeleken met een jaar geleden?	1 ☐ Veel beter nu dan een jaar geleden<br>2 ☐ Wat beter nu dan een jaar geleden<br>3 ☐ Ongeveer hetzelfde als een jaar geleden<br>4 ☐ Wat slechter nu dan een jaar geleden<br>5 ☐ Veel slechter nu dan een jaar geleden

De volgende vragen gaan over de bezigheden die u misschien hebt op een doorsnee dag. Wordt u op dit moment beperkt door uw gezondheid bij deze bezigheden? Zo ja, in welke mate?		Ja, ernstig beperkt	Ja, enigszins beperkt	Nee, helemaal niet beperkt
3a.	Forse inspanning, zoals hardlopen, tillen van zware voorwerpen, een veeleisende sport beoefenen	1 ☐	2 ☐	3 ☐
3b.	Matige inspanning, zoals een tafel verplaatsen, stofzuigen, zwemmen of fietsen	1 ☐	2 ☐	3 ☐
3c.	Boodschappen dragen of tillen	1 ☐	2 ☐	3 ☐
3d.	Een paar trappen oplopen	1 ☐	2 ☐	3 ☐
3e.	Één trap oplopen	1 ☐	2 ☐	3 ☐
3f.	Bukken, knielen of hurken	1 ☐	2 ☐	3 ☐
3g.	Meer dan één kilometer lopen	1 ☐	2 ☐	3 ☐
3h.	Een paar honderd meter lopen	1 ☐	2 ☐	3 ☐
3i.	Ongeveer honderd meter lopen	1 ☐	2 ☐	3 ☐
3j.	Uzelf wassen of aankleden	1 ☐	2 ☐	3 ☐

De volgende vragen gaan over de ***afgelopen 4 weken****. Heeft u één van de volgende problemen gehad bij uw werk of dagelijkse bezigheden ten gevolge van uw lichamelijke gezondheid?*

4a.	U besteedde minder tijd aan werk of andere bezigheden	1 ☐ Ja	0 ☐ Nee
4b.	U heeft minder bereikt dan u zou willen	1 ☐ Ja	0 ☐ Nee
4c.	U was beperkt in het soort werk of andere bezigheden	1 ☐ Ja	0 ☐ Nee
4d.	U had moeite om uw werk of andere bezigheden uit te voeren (kostte bijvoorbeeld extra inspanning)	1 ☐ Ja	0 ☐ Nee

De volgende vragen gaan over de ***afgelopen 4 weken****. Heeft u één van de volgende problemen gehad bij uw werk of dagelijkse bezigheden ten gevolge van emotionele problemen?*

5a.	U besteedde minder tijd aan werk of andere bezigheden	1 ☐ Ja	0 ☐ Nee
5b.	U heeft minder bereikt dan u zou willen	1 ☐ Ja	0 ☐ Nee
5c.	U deed uw werk of andere bezigheden niet zo zorgvuldig als gewoonlijk	1 ☐ Ja	0 ☐ Nee

6. In hoeverre hebben uw lichamelijke gezondheid of emotionele problemen u gedurende de **afgelopen 4 weken** gehinderd in uw normale omgang met familie, vrienden, buren, of bij activiteiten in groepsverband?

   1 ☐ Helemaal niet
   2 ☐ Enigszins
   3 ☐ Nogal
   4 ☐ Veel
   5 ☐ Heel erg veel

7.	Hoeveel lichamelijke pijn heeft u gedurende de **afgelopen 4 weken** gehad?	1 ☐ Geen<br>2 ☐ Zeer licht<br>3 ☐ Licht<br>4 ☐ Matig<br>5 ☐ Ernstig<br>6 ☐ Heel ernstig
8.	In welke mate bent u gedurende de **afgelopen 4 weken** door pijn gehinderd in uw normale werk (zowel werk buitenshuis als huishoudelijk werk)?	1 ☐ Helemaal niet<br>2 ☐ Enigszins<br>3 ☐ Nogal<br>4 ☐ Veel<br>5 ☐ Heel erg veel

De volgende vragen gaan over hoe u zich voelt en hoe het met u ging gedurende de afgelopen 4 weken. Wilt u alstublieft bij elke vraag het antwoord geven dat het best benadert hoe u zich voelde.
*Hoe vaak gedurende de **afgelopen 4 weken**…*

		Altijd	Meestal	Vaak	Soms	Zelden	Nooit
9a.	Voelde u zich levenslustig?	1 ☐	2 ☐	3 ☐	4 ☐	5 ☐	6 ☐
9b.	Was u erg zenuwachtig?	1 ☐	2 ☐	3 ☐	4 ☐	5 ☐	6 ☐
9c.	Zat u zo in de put dat niets u kon opvrolijken?	1 ☐	2 ☐	3 ☐	4 ☐	5 ☐	6 ☐
9d.	Voelde u zich rustig en tevreden?	1 ☐	2 ☐	3 ☐	4 ☐	5 ☐	6 ☐
9e.	Had u veel energie?	1 ☐	2 ☐	3 ☐	4 ☐	5 ☐	6 ☐
9f.	Voelde u zich somber en neerslachtig?	1 ☐	2 ☐	3 ☐	4 ☐	5 ☐	6 ☐
9g.	Voelde u zich uitgeput?	1 ☐	2 ☐	3 ☐	4 ☐	5 ☐	6 ☐
9h.	Was u een gelukkig mens?	1 ☐	2 ☐	3 ☐	4 ☐	5 ☐	6 ☐
9i.	Voelde u zich moe?	1 ☐	2 ☐	3 ☐	4 ☐	5 ☐	6 ☐

10. Hoe vaak hebben uw lichamelijke gezondheid of emotionele problemen u gedurende de afgelopen 4 weken gehinderd bij uw sociale activiteiten (zoals vrienden of familie bezoeken, enz.)?

1 ☐ Altijd 2 ☐ Meestal 3 ☐ Soms 4 ☐ Zelden 5 ☐ Nooit

Geef van de volgende uitspraken alstublieft aan hoe juist of onjuist deze voor u zijn.

		Helemaal juist	Grotendeels juist	Weet ik niet	Grotendeels onjuist	Helemaal onjuist
11a.	Ik lijk gemakkelijker ziek te worden dan andere mensen	1 ☐	2 ☐	3 ☐	4 ☐	5 ☐
11b.	Ik ben even gezond als andere mensen die ik ken	1 ☐	2 ☐	3 ☐	4 ☐	5 ☐
11c.	Ik verwacht dat mijn gezondheid achteruit zal gaan	1 ☐	2 ☐	3 ☐	4 ☐	5 ☐
11d.	Mijn gezondheid is uitstekend	1 ☐	2 ☐	3 ☐	4 ☐	5 ☐

Deel 1D: Uw nierziekte

Geef van de volgende uitspraken alstublieft aan hoe juist of onjuist deze voor u zijn.		Helemaal juist	Groten-deels juist	Weet ik niet	Groten-deels onjuist	Helemaal onjuist
12a.	Mijn nierziekte grijpt teveel in mijn leven in	1 ☐	2 ☐	3 ☐	4 ☐	5 ☐
12b.	Het omgaan met mijn nierziekte kost mij teveel tijd	1 ☐	2 ☐	3 ☐	4 ☐	5 ☐
12c.	Het omgaan met mijn nierziekte frustreert mij	1 ☐	2 ☐	3 ☐	4 ☐	5 ☐
12d.	Ik heb het gevoel dat ik mijn familie tot last ben door mijn nierziekte	1 ☐	2 ☐	3 ☐	4 ☐	5 ☐

*De volgende vragen gaan over hoe u zich voelt en hoe het met u ging gedurende de afgelopen 4 weken. Wilt u alstublieft bij elke vraag het antwoord geven dat het best benadert hoe u zich voelde. Hoe vaak gedurende de **afgelopen 4 weken**…*		Altijd	Meestal	Vaak	Soms	Zelden	Nooit
13a.	Zonderde u zich af van de mensen om u heen?	1 ☐	2 ☐	3 ☐	4 ☐	5 ☐	6 ☐
13b.	Reageerde u traag op dingen die werden gezegd of gedaan?	1 ☐	2 ☐	3 ☐	4 ☐	5 ☐	6 ☐
13c.	Gedroeg u zich geïrriteerd tegenover de mensen om u heen?	1 ☐	2 ☐	3 ☐	4 ☐	5 ☐	6 ☐
13d.	Had u moeite zich te concentreren of na te denken?	1 ☐	2 ☐	3 ☐	4 ☐	5 ☐	6 ☐
13e.	Kon u goed opschieten met andere mensen?	1 ☐	2 ☐	3 ☐	4 ☐	5 ☐	6 ☐
13f.	Raakte u in de war?	1 ☐	2 ☐	3 ☐	4 ☐	5 ☐	6 ☐

*Hoeveel last had u gedurende de **afgelopen 4 weken** van het volgende?*		Helemaal geen last	Beetje last	Nogal wat last	Veel last	Heel erg veel last
14a.	Pijnlijke spieren?	1 ☐	2 ☐	3 ☐	4 ☐	5 ☐
14b.	Pijn op de borst?	1 ☐	2 ☐	3 ☐	4 ☐	5 ☐
14c.	Kramp?	1 ☐	2 ☐	3 ☐	4 ☐	5 ☐
14d.	Jeuk op de huid?	1 ☐	2 ☐	3 ☐	4 ☐	5 ☐
14e.	Droge huid?	1 ☐	2 ☐	3 ☐	4 ☐	5 ☐
14f.	Kortademigheid?	1 ☐	2 ☐	3 ☐	4 ☐	5 ☐
14g.	Flauwte of duizeligheid?	1 ☐	2 ☐	3 ☐	4 ☐	5 ☐

14h.	Gebrek aan eetlust?		1 ☐	2 ☐	3 ☐	4 ☐	5 ☐
14i.	Helemaal uitgeput?		1 ☐	2 ☐	3 ☐	4 ☐	5 ☐
14j.	Gevoelloosheid in handen of voeten?		1 ☐	2 ☐	3 ☐	4 ☐	5 ☐
14k.	Misselijkheid of opspelende maag?		1 ☐	2 ☐	3 ☐	4 ☐	5 ☐

Sommige mensen hebben last van hun nierziekte in hun dagelijkse leven, andere mensen niet. Hoeveel last heeft u van uw nierziekte op elk van de volgende gebieden?			Helemaal geen last	Beetje last	Nogal wat last	Veel last	Heel erg veel last
15a.	Vochtbeperking?	0 ☐ n.v.t.	1 ☐	2 ☐	3 ☐	4 ☐	5 ☐
15b.	Dieetbeperking ?	0 ☐ n.v.t.	1 ☐	2 ☐	3 ☐	4 ☐	5 ☐
15c.	In staat zijn klusjes rond het huis te doen?		1 ☐	2 ☐	3 ☐	4 ☐	5 ☐
15d.	In staat zijn te reizen?		1 ☐	2 ☐	3 ☐	4 ☐	5 ☐
15e.	Afhankelijk zijn van artsen en andere zorgverleners?		1 ☐	2 ☐	3 ☐	4 ☐	5 ☐
15f.	Stress of zorgen veroorzaakt door uw nierziekte?		1 ☐	2 ☐	3 ☐	4 ☐	5 ☐
15g.	Uw seksleven?		1 ☐	2 ☐	3 ☐	4 ☐	5 ☐
15h.	Uw uiterlijk?		1 ☐	2 ☐	3 ☐	4 ☐	5 ☐

De volgende twee vragen zijn persoonlijk en gaan over uw seksuele activiteiten. Uw antwoorden zijn belangrijk om te kunnen begrijpen hoe een nierziekte het leven beïnvloedt. Hoe moeilijk vond u het volgende gedurende de ***afgelopen 4 weken****?*		Geen problem-en	Weinig problem-en	Nogal wat problem-en	Ernstige problem-en	Zeer ernstige problem-en
16a.	Genieten van de seks?	1 ☐	2 ☐	3 ☐	4 ☐	5 ☐
16b.	Seksueel opgewonden raken?	1 ☐	2 ☐	3 ☐	4 ☐	5 ☐

Geef bij de volgende vraag een score voor hoe goed u slaapt; een 0 betekent "heel erg slecht" en een 10 betekent "heel erg goed". Als u vindt dat uw slaap halverwege tussen "heel erg slecht"en "heel erg goed"ligt, zet dan een kruisje in het vakje rechts naast nummer 5. Als u vindt dat uw slaap een punt beter is dan 5, zet dan een kruisje in het vakje rechts naast nummer 6. Als u vindt dat uw slaap een punt slechter is dan 5, zet dan een kruisje rechts naast 4, enzovoorts.

Welke score geeft u voor hoe goed u in het algemeen slaapt, op een schaal van 0 tot 10?

17. Heel erg slecht — Heel erg goed

0 ☐ 1 ☐ 2 ☐ 3 ☐ 4 ☐ 5 ☐ 6 ☐ 7 ☐ 8 ☐ 9 ☐ 10 ☐

*Hoe vaak gedurende de **afgelopen 4 weken**…*		Altijd	Meestal	Vaak	Soms	Zelden	Nooit
18a.	Werd u ’s nachts wakker en had u moeite weer in slaap te vallen?	1 ☐	2 ☐	3 ☐	4 ☐	5 ☐	6 ☐
18b.	Kreeg u voldoende slaap?	1 ☐	2 ☐	3 ☐	4 ☐	5 ☐	6 ☐
18c.	Had u moeite om overdag wakker te blijven?	1 ☐	2 ☐	3 ☐	4 ☐	5 ☐	6 ☐

Wat betreft uw familie en vrienden, hoe tevreden bent u met...		Zeer ontevreden	Enigszins ontevreden	Enigszins tevreden	Zeer tevreden
19a.	De hoeveelheid tijd die u met familie en vrienden kunt doorbrengen?	1 ☐	2 ☐	3 ☐	4 ☐
19b.	De steun die u krijgt van familie en vrienden?	1 ☐	2 ☐	3 ☐	4 ☐
20a.	Verrichtte u gedurende de **afgelopen 4 weken** betaal werk?	1 ☐ Ja	0 ☐ Nee		
20b.	Belemmert uw gezondheid het verrichten van betaald werk?	1 ☐ Ja	0 ☐ Nee		

Hoe beoordeelt u uw gezondheid in het algemeen?

Hierbij is 0 het “slechts denkbaar” wat gelijk staat aan even slecht of nog slechter dan dood zijn.

21. Slechtst denkbaar — Best denkbaar

0	1	2	3	4	5	6	7	8	9	10
☐	☐	☐	☐	☐	☐	☐	☐	☐	☐	☐

Deel 2: Gezondheid <u>voor de pijnbehandeling</u>

Deel 2A: Pijnklachten

1.	Wel eens nierpijn voor pijnbehandeling?	1 ☐ Ja	0 ☐ Nee
1b.	Zo ja, waar?	1 ☐ Links	0 ☐ Rechts
1c.	Zo ja, frequentie nierpijn per dag?		__________________keer
1d.	Zo ja, frequentie nierpijn per week?		__________________keer
1e.	Zo ja, ooit opgenomen vanwege nierpijn?	1 ☐ Ja	0 ☐ Nee
1f.	Gemiddelde pijn score nieren (VAS score) gemiddeld voor de pijnbehandeling? (0 – 100)		_________________score
1g.	Maximale pijn score nieren (VAS score) voor de pijnbehandeling? (0 – 100)		_________________score
1h.	Minimale pijn score nieren (VAS score) voor de pijnbehandeling? (0 – 100)		_________________score

Medicatie

1.	Gebruikte u voor pijnbehandeling pijnmedicatie?	1 ☐ Ja	0 ☐ Nee
1b.	Zo ja, welke?	1 ☐ Paracetamol	
		2 ☐ NSAID’s (o.a. ibuprofen, naproxen, diclofenac)	
		3 ☐ Morfinepreparaten	
		4 ☐ Anders, namelijk______________________	
2.	Graag alle medicatie met doseringen noteren:		

____

____

____

____

Deel 2B: Patiënt geestelijke gezondheid - Patient Health Questionnaire (PHQ-9)

	Hoe vaak had u voor de pijnbehandeling last van één of meer van de volgende problemen?	Helemaal niet	Verschei-dene dagen	Meer dan de helft van de dagen	Bijna elke dag
1.	Weinig interesse of plezier om dingen te doen	0 ☐	1 ☐	2 ☐	3 ☐
2.	Zich neerslachtig, gedeprimeerd of hopeloos voelen	0 ☐	1 ☐	2 ☐	3 ☐
3.	Moeilijk inslapen, moeilijk doorslapen of teveel slapen	0 ☐	1 ☐	2 ☐	3 ☐
4.	Zich moe voelen of gebrek aan energie hebben	0 ☐	1 ☐	2 ☐	3 ☐
5.	Weinig eetlust of overmatig eten	0 ☐	1 ☐	2 ☐	3 ☐
6.	Een slecht gevoel hebben over uzelf – of het gevoel hebben dat u een mislukking bent of het gevoel dat u zichzelf of uw familie teleurgesteld hebt	0 ☐	1 ☐	2 ☐	3 ☐
7.	Problemen om u te concentreren, bijvoorbeeld om de krant te lezen of om tv te kijken	0 ☐	1 ☐	2 ☐	3 ☐
8.	Zo traag bewegen of zo langzaam spreken dat andere mensen dit opgemerkt kunnen hebben? Of het tegenovergestelde, zo zenuwachtig of rusteloos zijn dat u veel meer bewoog dan gebruikelijk	0 ☐	1 ☐	2 ☐	3 ☐
9.	De gedachte dat u beter dood zou kunnen zijn of de gedachte uzelf op een bepaalde manier pijn te doen	0 ☐	1 ☐	2 ☐	3 ☐
	**For office coding**	__*0*__	+ ______	+ ______	+ ______
					**Total score:** ________

10. Als u **enig** probleem hebt aangekruist, hoe **moeilijk** maakten deze problemen het dan voor u om uw werk of uw taken in en om het huis te doen, of om met andere mensen om te gaan?

1 ☐ Helemaal niet moeilijk   2 ☐ Enigszins moeilijk   3 ☐ Erg moeilijk   4 ☐ Extreem moeilijk

Deel 2C: Algehele gezondheid en kwaliteit van leven (SF-36)

Uw gezondheid voor de pijnbehandeling

1.	Hoe zou u over het algemeen uw gezondheid noemen?	1 ☐ Uitstekend<br>2 ☐ Zeer goed<br>3 ☐ Goed<br>4 ☐ Matig<br>5 ☐ Slecht
2.	Hoe beoordeelde u voor de pijnbehandeling uw gezondheid in het algemeen, vergeleken met een jaar eerder?	1 ☐ Veel beter nu dan een jaar eerder<br>2 ☐ Wat beter nu dan een jaar eerder<br>3 ☐ Ongeveer hetzelfde als een jaar eerder<br>4 ☐ Wat slechter nu dan een jaar eerder<br>5 ☐ Veel slechter nu dan een jaar eerder

	De volgende vragen gaan over de bezigheden die u misschien hebt op een doorsnee dag. Werd u voor de pijnbehandeling beperkt door uw gezondheid bij deze bezigheden? Zo ja, in welke mate?	Ja, ernstig beperkt	Ja, enigszins beperkt	Nee, helemaal niet beperkt
3a.	Forse inspanning, zoals hardlopen, tillen van zware voorwerpen, een veeleisende sport beoefenen	1 ☐	2 ☐	3 ☐
3b.	Matige inspanning, zoals een tafel verplaatsen, stofzuigen, zwemmen of fietsen	1 ☐	2 ☐	3 ☐
3c.	Boodschappen dragen of tillen	1 ☐	2 ☐	3 ☐
3d.	Een paar trappen oplopen	1 ☐	2 ☐	3 ☐
3e.	Één trap oplopen	1 ☐	2 ☐	3 ☐
3f.	Bukken, knielen of hurken	1 ☐	2 ☐	3 ☐
3g.	Meer dan één kilometer lopen	1 ☐	2 ☐	3 ☐
3h.	Een paar honderd meter lopen	1 ☐	2 ☐	3 ☐
3i.	Ongeveer honderd meter lopen	1 ☐	2 ☐	3 ☐

3j.	Uzelf wassen of aankleden	1 ☐	2 ☐	3 ☐

De volgende vragen gaan over de periode ***voor de pijnbehandeling****. Heeft u één van de volgende problemen gehad bij uw werk of dagelijkse bezigheden ten gevolge van uw lichamelijke gezondheid?*

4a.	U besteedde minder tijd aan werk of andere bezigheden	1 ☐ Ja	0 ☐ Nee
4b.	U had minder bereikt dan u zou willen	1 ☐ Ja	0 ☐ Nee
4c.	U was beperkt in het soort werk of andere bezigheden	1 ☐ Ja	0 ☐ Nee
4d.	U had moeite om uw werk of andere bezigheden uit te voeren (kostte bijvoorbeeld extra inspanning)	1 ☐ Ja	0 ☐ Nee

De volgende vragen gaan over de periode ***voor de pijnbehandeling****. Heeft u één van de volgende problemen gehad bij uw werk of dagelijkse bezigheden ten gevolge van emotionele problemen?*

5a.	U besteedde minder tijd aan werk of andere bezigheden	1 ☐ Ja	0 ☐ Nee
5b.	U had minder bereikt dan u zou willen	1 ☐ Ja	0 ☐ Nee
5c.	U deed uw werk of andere bezigheden niet zo zorgvuldig als gewoonlijk	1 ☐ Ja	0 ☐ Nee

6.	In hoeverre hadden uw lichamelijke gezondheid of emotionele problemen u **voor de pijnbehandeling** gehinderd in uw normale omgang met familie, vrienden, buren, of bij activiteiten in groepsverband?	1 ☐ Helemaal niet<br>2 ☐ Enigszins<br>3 ☐ Nogal<br>4 ☐ Veel<br>5 ☐ Heel erg veel
7.	Hoeveel lichamelijke pijn had u in de periode **voor de pijnbehandeling**?	1 ☐ Geen<br>2 ☐ Zeer licht<br>3 ☐ Licht<br>4 ☐ Matig<br>5 ☐ Ernstig<br>6 ☐ Heel ernstig
8.	In welke mate was u **voor de pijnbehandeling** door pijn gehinderd in uw normale werk (zowel werk buitenshuis als huishoudelijk werk)?	1 ☐ Helemaal niet<br>2 ☐ Enigszins<br>3 ☐ Nogal<br>4 ☐ Veel<br>5 ☐ Heel erg veel

De volgende vragen gaan over hoe u zich voelt en hoe het met u ging <u>voor de pijnbehandeling</u>. Wilt u alstublieft bij elke vraag het antwoord geven dat het best benadert hoe u zich voelde.<br>*Hoe vaak in de 4 weken <u>voor de pijnbehandeling</u>*		Altijd	Meestal	Vaak	Soms	Zelden	Nooit
9a.	Voelde u zich levenslustig?	1 ☐	2 ☐	3 ☐	4 ☐	5 ☐	6 ☐
9b.	Was u erg zenuwachtig?	1 ☐	2 ☐	3 ☐	4 ☐	5 ☐	6 ☐
9c.	Zat u zo in de put dat niets u kon opvrolijken?	1 ☐	2 ☐	3 ☐	4 ☐	5 ☐	6 ☐
9d.	Voelde u zich rustig en tevreden?	1 ☐	2 ☐	3 ☐	4 ☐	5 ☐	6 ☐
9e.	Had u veel energie?	1 ☐	2 ☐	3 ☐	4 ☐	5 ☐	6 ☐
9f.	Voelde u zich somber en neerslachtig?	1 ☐	2 ☐	3 ☐	4 ☐	5 ☐	6 ☐
9g.	Voelde u zich uitgeput?	1 ☐	2 ☐	3 ☐	4 ☐	5 ☐	6 ☐
9h.	Was u een gelukkig mens?	1 ☐	2 ☐	3 ☐	4 ☐	5 ☐	6 ☐
9i.	Voelde u zich moe?	1 ☐	2 ☐	3 ☐	4 ☐	5 ☐	6 ☐

10. Hoe vaak hebben uw <u>lichamelijke gezondheid of emotionele problemen</u> u voor de pijnbehandeling gehinderd bij uw sociale activiteiten (zoals vrienden of familie bezoeken, enz.)?

1 ☐ Altijd   2 ☐ Meestal   3 ☐ Soms   4 ☐ Zelden   5 ☐ Nooit

Geef van de volgende uitspraken alstublieft aan hoe <u>juist</u> of <u>onjuist</u> deze voor u zijn.		Helema al juist	Groten-deels juist	Weet ik niet	Groten-deels onjuist	Helemaal onjuist
11a.	Ik leek gemakkelijker ziek te worden dan andere mensen	1 ☐	2 ☐	3 ☐	4 ☐	5 ☐
11b.	Ik was even gezond als andere mensen die ik ken	1 ☐	2 ☐	3 ☐	4 ☐	5 ☐
11c.	Ik verwachtte dat mijn gezondheid achteruit zou gaan	1 ☐	2 ☐	3 ☐	4 ☐	5 ☐
11d.	Mijn gezondheid was uitstekend	1 ☐	2 ☐	3 ☐	4 ☐	5 ☐

Deel 2D: Uw nierziekte voor de pijnbehandeling

Geef van de volgende uitspraken alstublieft aan hoe <u>juist</u> of <u>onjuist</u> deze voor u zijn.		Helemaal juist	Groten -deels juist	Weet ik niet	Groten-deels onjuist	Helemaal onjuist
12a.	Mijn nierziekte greep teveel in mijn leven in	1 ☐	2 ☐	3 ☐	4 ☐	5 ☐
12b.	Het omgaan met mijn nierziekte koste mij teveel tijd	1 ☐	2 ☐	3 ☐	4 ☐	5 ☐

12c.	Het omgaan met mijn nierziekte frustreerde mij	1 ☐	2 ☐	3 ☐	4 ☐	5 ☐	
12d.	Ik had het gevoel dat ik mijn familie tot last was door mijn nierziekte	1 ☐	2 ☐	3 ☐	4 ☐	5 ☐	

De volgende vragen gaan over hoe u zich voelt en hoe het met u ging <u>voor de pijnbehandeling</u>. Wilt u alstublieft bij elke vraag het antwoord geven dat het best benadert hoe u zich voelde.<br>*Hoe vaak **<u>in de periode voor de pijnbehandeling…</u>***		Altijd	Meestal	Vaak	Soms	Zelden	Nooit
13a.	Zonderde u zich af van de mensen om u heen?	1 ☐	2 ☐	3 ☐	4 ☐	5 ☐	6 ☐
13b.	Reageerde u traag op dingen die werden gezegd of gedaan?	1 ☐	2 ☐	3 ☐	4 ☐	5 ☐	6 ☐
13c.	Gedroeg u zich geïrriteerd tegenover de mensen om u heen?	1 ☐	2 ☐	3 ☐	4 ☐	5 ☐	6 ☐
13d.	Had u moeite zich te concentreren of na te denken?	1 ☐	2 ☐	3 ☐	4 ☐	5 ☐	6 ☐
13e.	Kon u goed opschieten met andere mensen?	1 ☐	2 ☐	3 ☐	4 ☐	5 ☐	6 ☐
13f.	Raakte u in de war?	1 ☐	2 ☐	3 ☐	4 ☐	5 ☐	6 ☐

*Hoeveel last had u **<u>voor de pijnbehandeling</u>** van het volgende?*		Helemaal geen last	Beetje last	Nogal wat last	Veel last	Heel erg veel last
14a.	Pijnlijke spieren?	1 ☐	2 ☐	3 ☐	4 ☐	5 ☐
14b.	Pijn op de borst?	1 ☐	2 ☐	3 ☐	4 ☐	5 ☐
14c.	Kramp?	1 ☐	2 ☐	3 ☐	4 ☐	5 ☐
14d.	Jeuk op de huid?	1 ☐	2 ☐	3 ☐	4 ☐	5 ☐
14e.	Droge huid?	1 ☐	2 ☐	3 ☐	4 ☐	5 ☐
14f.	Kortademigheid?	1 ☐	2 ☐	3 ☐	4 ☐	5 ☐
14g.	Flauwte of duizeligheid?	1 ☐	2 ☐	3 ☐	4 ☐	5 ☐
14h.	Gebrek aan eetlust?	1 ☐	2 ☐	3 ☐	4 ☐	5 ☐
14i.	Helemaal uitgeput?	1 ☐	2 ☐	3 ☐	4 ☐	5 ☐
14j.	Gevoelloosheid in handen of voeten?	1 ☐	2 ☐	3 ☐	4 ☐	5 ☐
14k.	Misselijkheid of opspelende maag?	1 ☐	2 ☐	3 ☐	4 ☐	5 ☐

Sommige mensen hebben last van hun nierziekte in hun dagelijkse leven, andere mensen niet. Hoeveel <u>last</u> had u van uw nierziekte op elk van de volgende gebieden?		Helemaal geen last	Beetje last	Nogal wat last	Veel last	Heel erg veel last
15a. Vochtbeperking?	0 ☐ n.v.t.	1 ☐	2 ☐	3 ☐	4 ☐	5 ☐
15b. Dieetbeperking ?	0 ☐ n.v.t.	1 ☐	2 ☐	3 ☐	4 ☐	5 ☐
15c. In staat zijn klusjes rond het huis te doen?		1 ☐	2 ☐	3 ☐	4 ☐	5 ☐
15d. In staat zijn te reizen?		1 ☐	2 ☐	3 ☐	4 ☐	5 ☐
15e. Afhankelijk zijn van artsen en andere zorgverleners?		1 ☐	2 ☐	3 ☐	4 ☐	5 ☐
15f. Stress of zorgen veroorzaakt door uw nierziekte?		1 ☐	2 ☐	3 ☐	4 ☐	5 ☐
15g. Uw seksleven?		1 ☐	2 ☐	3 ☐	4 ☐	5 ☐
15h. Uw uiterlijk?		1 ☐	2 ☐	3 ☐	4 ☐	5 ☐

De volgende twee vragen zijn persoonlijk en gaan over uw seksuele activiteiten. Uw antwoorden zijn belangrijk om te kunnen begrijpen hoe een nierziekte het leven beïnvloedt. Hoe moeilijk vond u het volgende gedurende de ***<u>periode voor de pijnbehandeling</u>****?*	Geen problem-en	Weinig problem-en	Nogal wat problem-en	Ernstige problem-en	Zeer ernstige problem-en
16a. Genieten van de seks?	1 ☐	2 ☐	3 ☐	4 ☐	5 ☐
16b. Seksueel opgewonden raken?	1 ☐	2 ☐	3 ☐	4 ☐	5 ☐

Geef bij de volgende vraag een score voor hoe goed u sliep; een 0 betekent "heel erg slecht" en een 10 betekent "heel erg goed". Als u vindt dat uw slaap halverwege tussen "heel erg slecht" en "heel erg goed" lag, zet dan een kruisje in het vakje rechts naast nummer 5. Als u vindt dat uw slaap een punt beter was dan 5, zet dan een kruisje in het vakje rechts naast nummer 6. Als u vindt dat uw slaap een punt slechter was dan 5, zet dan een kruisje rechts naast 4, enzovoorts.

Welke score geeft u voor hoe goed u in het algemeen sliep, op een schaal van 0 tot 10?

17. Heel erg slecht — Heel erg goed

0 ☐ 1 ☐ 2 ☐ 3 ☐ 4 ☐ 5 ☐ 6 ☐ 7 ☐ 8 ☐ 9 ☐ 10 ☐

Hoe vaak gedurende de ***periode voor de pijnbehandeling****…*	Altijd	Meestal	Vaak	Soms	Zelden	Nooit
18a. Werd u 's nachts wakker en had u moeite weer in slaap te vallen?	1 ☐	2 ☐	3 ☐	4 ☐	5 ☐	6 ☐
18b. Kreeg u voldoende slaap?	1 ☐	2 ☐	3 ☐	4 ☐	5 ☐	6 ☐
18c. Had u moeite om overdag wakker te blijven?	1 ☐	2 ☐	3 ☐	4 ☐	5 ☐	6 ☐

Wat betreft uw <u>familie en vrienden</u>, hoe tevreden was u met...	Zeer ontevreden	Enigszins ontevreden	Enigszins tevreden	Zeer tevreden
19a. De hoeveelheid tijd die u met familie en vrienden kon doorbrengen?	1 ☐	2 ☐	3 ☐	4 ☐
19b. De steun die u kreeg van familie en vrienden?	1 ☐	2 ☐	3 ☐	4 ☐

20a.	Verrichtte u gedurende de **periode voor de pijnbehandeling** betaal werk?	1 ☐ Ja	0 ☐ Nee
20b.	Belemmerde uw gezondheid het verrichten van betaald werk?	1 ☐ Ja	0 ☐ Nee

Hoe beoordeelde u uw gezondheid in het algemeen?

Hierbij is 0 het "slechts denkbaar" wat gelijk staat aan even slecht of nog slechter dan dood zijn.

21.	Slechtst denkbaar										Best denkbaar
	0 ☐	1 ☐	2 ☐	3 ☐	4 ☐	5 ☐	6 ☐	7 ☐	8 ☐	9 ☐	10 ☐

Deel 3: Gezondheid <u>6 weken na de pijnbehandeling</u>

Deel 3A: Pijnklachten

1.	Wel eens nierpijn 6 weken na de pijnbehandeling?	1 ☐ Ja	0 ☐ Nee
1b.	Zo ja, waar?	1 ☐ Links	0 ☐ Rechts
1c.	Zo ja, frequentie nierpijn per dag?		__________________keer
1d.	Zo ja, frequentie nierpijn per week?		__________________keer
1e.	Zo ja, ooit opgenomen vanwege nierpijn?	1 ☐ Ja	0 ☐ Nee
1f.	Gemiddelde pijn score nieren (VAS score) gemiddeld 6 weken na de pijnbehandeling? (0 – 100)		_________________score
1g.	Maximale pijn score nieren (VAS score) 6 weken na de pijnbehandeling? (0 – 100)		_________________score
1h.	Minimale pijn score nieren (VAS score) 6 weken na de pijnbehandeling? (0 – 100)		_________________score

Medicatie

1.	Gebruikte u 6 weken na de pijnbehandeling pijnmedicatie?	1 ☐ Ja	0 ☐ Nee
1b.	Zo ja, welke?	1 ☐ Paracetamol	
		2 ☐ NSAID's (o.a. ibuprofen, naproxen, diclofenac)	
		3 ☐ Morfinepreparaten	
		4 ☐ Anders, namelijk_______________________	
2.	Graag alle medicatie met doseringen noteren:		

____

____

____

____

Deel 3B: Patiënt geestelijke gezondheid - Patient Health Questionnaire (PHQ-9)

Hoe vaak had u 6 weken na de pijnbehandeling last van één of meer van de volgende problemen?	Helemaal niet	Verschei-dene dagen	Meer dan de helft van de dagen	Bijna elke dag
1. Weinig interesse of plezier om dingen te doen	0 ☐	1 ☐	2 ☐	3 ☐
2. Zich neerslachtig, gedeprimeerd of hopeloos voelen	0 ☐	1 ☐	2 ☐	3 ☐
3. Moeilijk inslapen, moeilijk doorslapen of teveel slapen	0 ☐	1 ☐	2 ☐	3 ☐
4. Zich moe voelen of gebrek aan energie hebben	0 ☐	1 ☐	2 ☐	3 ☐
5. Weinig eetlust of overmatig eten	0 ☐	1 ☐	2 ☐	3 ☐
6. Een slecht gevoel hebben over uzelf – of het gevoel hebben dat u een mislukking bent of het gevoel dat u zichzelf of uw familie teleurgesteld hebt	0 ☐	1 ☐	2 ☐	3 ☐
7. Problemen om u te concentreren, bijvoorbeeld om de krant te lezen of om tv te kijken	0 ☐	1 ☐	2 ☐	3 ☐
8. Zo traag bewegen of zo langzaam spreken dat andere mensen dit opgemerkt kunnen hebben? Of het tegenovergestelde, zo zenuwachtig of rusteloos zijn dat u veel meer bewoog dan gebruikelijk	0 ☐	1 ☐	2 ☐	3 ☐
9. De gedachte dat u beter dood zou kunnen zijn of de gedachte uzelf op een bepaalde manier pijn te doen	0 ☐	1 ☐	2 ☐	3 ☐
**For office coding**	*0*	+ ______	+ ______	+ ______

Total score: ________

10. Als u **enig** probleem hebt aangekruist, hoe **moeilijk** maakten deze problemen het dan voor u om uw werk of uw taken in en om het huis te doen, of om met andere mensen om te gaan?

1 ☐ Helemaal niet moeilijk 2 ☐ Enigszins moeilijk 3 ☐ Erg moeilijk 4 ☐ Extreem moeilijk

Deel 3C: Algehele gezondheid en kwaliteit van leven (SF-36)

Uw gezondheid 6 weken na de pijnbehandeling

1. Hoe zou u 6 weken na de pijnbehandeling over het algemeen uw gezondheid noemen?
   - 1 ☐ Uitstekend
   - 2 ☐ Zeer goed
   - 3 ☐ Goed
   - 4 ☐ Matig
   - 5 ☐ Slecht

2. Hoe beoordeelde u 6 weken na de pijnbehandeling uw gezondheid in het algemeen, vergeleken met een jaar eerder?
   - 1 ☐ Veel beter nu dan een jaar eerder
   - 2 ☐ Wat beter nu dan een jaar geleden
   - 3 ☐ Ongeveer hetzelfde als een jaar geleden
   - 4 ☐ Wat slechter nu dan een jaar geleden
   - 5 ☐ Veel slechter nu dan een jaar geleden

	De volgende vragen gaan over de bezigheden die u misschien hebt op een doorsnee dag. Werd u 6 weken na de pijnbehandeling beperkt door uw gezondheid bij deze bezigheden? Zo ja, in welke mate?	Ja, ernstig beperkt	Ja, enigszins beperkt	Nee, helemaal niet beperkt
3a.	Forse inspanning, zoals hardlopen, tillen van zware voorwerpen, een veeleisende sport beoefenen	1 ☐	2 ☐	3 ☐
3b.	Matige inspanning, zoals een tafel verplaatsen, stofzuigen, zwemmen of fietsen	1 ☐	2 ☐	3 ☐
3c.	Boodschappen dragen of tillen	1 ☐	2 ☐	3 ☐
3d.	Een paar trappen oplopen	1 ☐	2 ☐	3 ☐
3e.	Één trap oplopen	1 ☐	2 ☐	3 ☐
3f.	Bukken, knielen of hurken	1 ☐	2 ☐	3 ☐
3g.	Meer dan één kilometer lopen	1 ☐	2 ☐	3 ☐
3h.	Een paar honderd meter lopen	1 ☐	2 ☐	3 ☐

3i.	<u>Ongeveer honderd meter</u> lopen	1 ☐	2 ☐	3 ☐
3j.	Uzelf wassen of aankleden	1 ☐	2 ☐	3 ☐

*De volgende vragen gaan over de periode **<u>6 weken na de pijnbehandeling</u>**. Heeft u één van de volgende problemen gehad bij uw werk of dagelijkse bezigheden <u>ten gevolge van uw lichamelijke gezondheid</u>?*

4a.	U besteedde <u>minder tijd</u> aan werk of andere bezigheden	1 ☐ Ja	0 ☐ Nee
4b.	<u>U had minder bereikt</u> dan u zou willen	1 ☐ Ja	0 ☐ Nee
4c.	U was beperkt in het <u>soort</u> werk of andere bezigheden	1 ☐ Ja	0 ☐ Nee
4d.	U had <u>moeite</u> om uw werk of andere bezigheden uit te voeren (kostte bijvoorbeeld extra inspanning)	1 ☐ Ja	0 ☐ Nee

*De volgende vragen gaan over de periode **<u>6 weken na de pijnbehandeling</u>**. Heeft u één van de volgende problemen gehad bij uw werk of dagelijkse bezigheden <u>ten gevolge van emotionele problemen</u>?*

5a.	U besteedde <u>minder tijd</u> aan werk of andere bezigheden	1 ☐ Ja	0 ☐ Nee
5b.	<u>U had minder bereikt</u> dan u zou willen	1 ☐ Ja	0 ☐ Nee
5c.	U deed uw werk of andere bezigheden niet zo <u>zorgvuldig</u> als gewoonlijk	1 ☐ Ja	0 ☐ Nee

6. In hoeverre hadden uw lichamelijke gezondheid of emotionele problemen u **<u>6 weken na de pijnbehandeling</u>** gehinderd in uw normale omgang met familie, vrienden, buren, of bij activiteiten in groepsverband?
   - 1 ☐ Helemaal niet
   - 2 ☐ Enigszins
   - 3 ☐ Nogal
   - 4 ☐ Veel
   - 5 ☐ Heel erg veel

7. Hoeveel <u>lichamelijke</u> pijn had u in de periode **<u>6 weken voor de pijnbehandeling</u>**?
   - 1 ☐ Geen
   - 2 ☐ Zeer licht
   - 3 ☐ Licht
   - 4 ☐ Matig
   - 5 ☐ Ernstig
   - 6 ☐ Heel ernstig

8. In welke mate was u **<u>6 weken na de pijnbehandeling</u>** door <u>pijn</u> gehinderd in uw normale werk (zowel werk buitenshuis als huishoudelijk werk)?
   - 1 ☐ Helemaal niet
   - 2 ☐ Enigszins
   - 3 ☐ Nogal
   - 4 ☐ Veel
   - 5 ☐ Heel erg veel

De volgende vragen gaan over hoe u zich voelt en hoe het met u ging <u>6 weken na de pijnbehandeling</u>. Wilt u alstublieft bij elke vraag het antwoord geven dat het best benadert hoe u zich voelde. Hoe vaak in de 4 weken <u>6 weken na de pijnbehandeling</u>		Altijd	Meestal	Vaak	Soms	Zelden	Nooit
9a.	Voelde u zich levenslustig?	1 ☐	2 ☐	3 ☐	4 ☐	5 ☐	6 ☐
9b.	Was u erg zenuwachtig?	1 ☐	2 ☐	3 ☐	4 ☐	5 ☐	6 ☐
9c.	Zat u zo in de put dat niets u kon opvrolijken?	1 ☐	2 ☐	3 ☐	4 ☐	5 ☐	6 ☐
9d.	Voelde u zich rustig en tevreden?	1 ☐	2 ☐	3 ☐	4 ☐	5 ☐	6 ☐
9e.	Had u veel energie?	1 ☐	2 ☐	3 ☐	4 ☐	5 ☐	6 ☐
9f.	Voelde u zich somber en neerslachtig?	1 ☐	2 ☐	3 ☐	4 ☐	5 ☐	6 ☐
9g.	Voelde u zich uitgeput?	1 ☐	2 ☐	3 ☐	4 ☐	5 ☐	6 ☐
9h.	Was u een gelukkig mens?	1 ☐	2 ☐	3 ☐	4 ☐	5 ☐	6 ☐
9i.	Voelde u zich moe?	1 ☐	2 ☐	3 ☐	4 ☐	5 ☐	6 ☐

10. Hoe vaak hebben uw <u>lichamelijke gezondheid of emotionele problemen</u> u 6 weken na de pijnbehandeling gehinderd bij uw sociale activiteiten (zoals vrienden of familie bezoeken, enz.)?

1 ☐ Altijd 2 ☐ Meestal 3 ☐ Soms 4 ☐ Zelden 5 ☐ Nooit

Geef van de volgende uitspraken alstublieft aan hoe <u>juist</u> of <u>onjuist</u> deze voor u zijn.		Helemaal juist	Grotendeels juist	Weet ik niet	Grotendeels onjuist	Helemaal onjuist
11a.	Ik leek gemakkelijker ziek te worden dan andere mensen	1 ☐	2 ☐	3 ☐	4 ☐	5 ☐
11b.	Ik was even gezond als andere mensen die ik ken	1 ☐	2 ☐	3 ☐	4 ☐	5 ☐
11c.	Ik verwachtte dat mijn gezondheid achteruit zou gaan	1 ☐	2 ☐	3 ☐	4 ☐	5 ☐
11d.	Mijn gezondheid was uitstekend	1 ☐	2 ☐	3 ☐	4 ☐	5 ☐

Deel 3D: Uw nierziekte 6 weken na de pijnbehandeling

Geef van de volgende uitspraken alstublieft aan hoe <u>juist</u> of <u>onjuist</u> deze voor u zijn.		Helemaal juist	Grotendeels juist	Weet ik niet	Grotendeels onjuist	Helemaal onjuist
12a.	Mijn nierziekte greep teveel in mijn leven in	1 ☐	2 ☐	3 ☐	4 ☐	5 ☐
12b.	Het omgaan met mijn nierziekte koste mij teveel tijd	1 ☐	2 ☐	3 ☐	4 ☐	5 ☐

12c.	Het omgaan met mijn nierziekte frustreerde mij	1 ☐	2 ☐	3 ☐	4 ☐	5 ☐
12d.	Ik had het gevoel dat ik mijn familie tot last was door mijn nierziekte	1 ☐	2 ☐	3 ☐	4 ☐	5 ☐

De volgende vragen gaan over hoe u zich voelt en hoe het met u ging *<u>6 weken na de pijnbehandeling</u>. Wilt u alstublieft bij elke vraag het antwoord geven dat het best benadert hoe u zich voelde.*<br>*Hoe vaak* ***<u>in de 6 weken na de pijnbehandeling…</u>***		Altijd	Meestal	Vaak	Soms	Zelden	Nooit
13a.	Zonderde u zich af van de mensen om u heen?	1 ☐	2 ☐	3 ☐	4 ☐	5 ☐	6 ☐
13b.	Reageerde u traag op dingen die werden gezegd of gedaan?	1 ☐	2 ☐	3 ☐	4 ☐	5 ☐	6 ☐
13c.	Gedroeg u zich geïrriteerd tegenover de mensen om u heen?	1 ☐	2 ☐	3 ☐	4 ☐	5 ☐	6 ☐
13d.	Had u moeite zich te concentreren of na te denken?	1 ☐	2 ☐	3 ☐	4 ☐	5 ☐	6 ☐
13e.	Kon u goed opschieten met andere mensen?	1 ☐	2 ☐	3 ☐	4 ☐	5 ☐	6 ☐
13f.	Raakte u in de war?	1 ☐	2 ☐	3 ☐	4 ☐	5 ☐	6 ☐

Hoeveel last had u ***<u>6 weken na de pijnbehandeling</u>*** *van het volgende?*		Helemaal geen last	Beetje last	Nogal wat last	Veel last	Heel erg veel last
14a.	Pijnlijke spieren?	1 ☐	2 ☐	3 ☐	4 ☐	5 ☐
14b.	Pijn op de borst?	1 ☐	2 ☐	3 ☐	4 ☐	5 ☐
14c.	Kramp?	1 ☐	2 ☐	3 ☐	4 ☐	5 ☐
14d.	Jeuk op de huid?	1 ☐	2 ☐	3 ☐	4 ☐	5 ☐
14e.	Droge huid?	1 ☐	2 ☐	3 ☐	4 ☐	5 ☐
14f.	Kortademigheid?	1 ☐	2 ☐	3 ☐	4 ☐	5 ☐
14g.	Flauwte of duizeligheid?	1 ☐	2 ☐	3 ☐	4 ☐	5 ☐
14h.	Gebrek aan eetlust?	1 ☐	2 ☐	3 ☐	4 ☐	5 ☐
14i.	Helemaal uitgeput?	1 ☐	2 ☐	3 ☐	4 ☐	5 ☐
14j.	Gevoelloosheid in handen of voeten?	1 ☐	2 ☐	3 ☐	4 ☐	5 ☐
14k.	Misselijkheid of opspelende maag?	1 ☐	2 ☐	3 ☐	4 ☐	5 ☐

	Sommige mensen hebben last van hun nierziekte in hun dagelijkse leven, andere mensen niet. Hoeveel <u>last</u> had u van uw nierziekte op elk van de volgende gebieden?		Helemaal geen last	Beetje last	Nogal wat last	Veel last	Heel erg veel last
15a.	Vochtbeperking?	0 ☐ n.v.t.	1 ☐	2 ☐	3 ☐	4 ☐	5 ☐
15b.	Dieetbeperking ?	0 ☐ n.v.t.	1 ☐	2 ☐	3 ☐	4 ☐	5 ☐
15c.	In staat zijn klusjes rond het huis te doen?		1 ☐	2 ☐	3 ☐	4 ☐	5 ☐
15d.	In staat zijn te reizen?		1 ☐	2 ☐	3 ☐	4 ☐	5 ☐
15e.	Afhankelijk zijn van artsen en andere zorgverleners?		1 ☐	2 ☐	3 ☐	4 ☐	5 ☐
15f.	Stress of zorgen veroorzaakt door uw nierziekte?		1 ☐	2 ☐	3 ☐	4 ☐	5 ☐
15g.	Uw seksleven?		1 ☐	2 ☐	3 ☐	4 ☐	5 ☐
15h.	Uw uiterlijk?		1 ☐	2 ☐	3 ☐	4 ☐	5 ☐

	*De volgende twee vragen zijn persoonlijk en gaan over uw seksuele activiteiten. Uw antwoorden zijn belangrijk om te kunnen begrijpen hoe een nierziekte het leven beïnvloedt. Hoe moeilijk vond u het volgende gedurende de **<u>6 weken na de pijnbehandeling</u>**?*	Geen problem-en	Weinig problem-en	Nogal wat problem-en	Ernstige problem-en	Zeer ernstige problem-en
16a.	Genieten van de seks?	1 ☐	2 ☐	3 ☐	4 ☐	5 ☐
16b.	Seksueel opgewonden raken?	1 ☐	2 ☐	3 ☐	4 ☐	5 ☐

Geef bij de volgende vraag een score voor hoe goed u sliep; een 0 betekent "heel erg slecht" en een 10 betekent "heel erg goed". Als u vindt dat uw slaap halverwege tussen "heel erg slecht" en "heel erg goed" lag, zet dan een kruisje in het vakje rechts naast nummer 5. Als u vindt dat uw slaap een punt beter was dan 5, zet dan een kruisje in het vakje rechts naast nummer 6. Als u vindt dat uw slaap een punt slechter was dan 5, zet dan een kruisje rechts naast 4, enzovoorts.

Welke score geeft u voor hoe goed u in het algemeen sliep, op een schaal van 0 tot 10?

17. Heel erg slecht — Heel erg goed

0 ☐	1 ☐	2 ☐	3 ☐	4 ☐	5 ☐	6 ☐	7 ☐	8 ☐	9 ☐	10 ☐

	*Hoe vaak gedurende de **6 weken na de pijnbehandeling**…*	Altijd	Meestal	Vaak	Soms	Zelden	Nooit
18a.	Werd u 's nachts wakker en had u moeite weer in slaap te vallen?	1 ☐	2 ☐	3 ☐	4 ☐	5 ☐	6 ☐
18b.	Kreeg u voldoende slaap?	1 ☐	2 ☐	3 ☐	4 ☐	5 ☐	6 ☐
18c.	Had u moeite om overdag wakker te blijven?	1 ☐	2 ☐	3 ☐	4 ☐	5 ☐	6 ☐

	Wat betreft uw <u>familie en vrienden</u>, hoe tevreden was u met...	Zeer ontevreden	Enigszins ontevreden	Enigszins tevreden	Zeer tevreden
19a.	De hoeveelheid tijd die u met familie en vrienden kon doorbrengen?	1 ☐	2 ☐	3 ☐	4 ☐
19b.	De steun die u kreeg van familie en vrienden?	1 ☐	2 ☐	3 ☐	4 ☐

20a.	Verrichtte u gedurende de **<u>6 weken na de pijnbehandeling</u>** betaal werk?	1 ☐ Ja	0 ☐ Nee
20b.	Belemmerde uw gezondheid het verrichten van betaald werk?	1 ☐ Ja	0 ☐ Nee

Hoe beoordeelde u <u>6 weken na de pijnbehandeling</u> uw gezondheid in het algemeen?

Hierbij is 0 het "slechts denkbaar" wat gelijk staat aan even slecht of nog slechter dan dood zijn.

21.	Slechtst denkbaar									Best denkbaar
0 ☐	1 ☐	2 ☐	3 ☐	4 ☐	5 ☐	6 ☐	7 ☐	8 ☐	9 ☐	10 ☐
